# Trades-offs between pollinator attraction and florivore defense maximize reproductive success in the self-incompatible *Rivea ornata* (Convolvulaceae)

**DOI:** 10.1186/s12862-024-02301-7

**Published:** 2024-08-30

**Authors:** Natthaphong Chitchak, Alyssa B. Stewart, Paweena Traiperm

**Affiliations:** Department of Plant Science, Faculty of Science, Mahidol University, Bangkok, 10400 Thailand

**Keywords:** Breeding system, Floral specialization, Plant defense, Sphingidae, Xenogamy

## Abstract

**Background:**

*Rivea ornata*, a rare species from the morning glory family, exhibits uncommon characteristics compared to other typical morning glories, including nocturnal flowers that fit the classic moth pollination syndrome. However, the accuracy of its predicted pollination syndrome and its mating system have never been assessed. Additionally, *R. ornata* flowers attract not only pollinators but also florivores, potentially reducing plant reproductive success. Therefore, this study examined two populations of *R. ornata* in Thailand and assessed traits related to pollinator attraction and reward, determined its mating system, identified floral visitors and effective pollinators, and investigated the effect of florivory on reproductive success.

**Results:**

*Rivea ornata* is highly fertile but self-incompatible and an obligate outcrosser, rendering it highly dependent on pollinators. Lepidopterans, particularly nocturnal hawk moths, were found to account for a significant proportion of all visits and were the sole effective pollinators of this plant species, in correspondence with its predicted pollination syndrome. Surprisingly, florivory did not significantly reduce reproductive success. This phenomenon may be explained by the strategies employed by *R. ornata*, which align with the optimal defense hypothesis and functional trade-offs. Specifically, *R. ornata* appears to invest resources in defending key floral structures while, simultaneously, guard ants are conspicuously absent from flowers, resulting in some florivore damage to non-vital floral organs but ensuring that pollinators are not deterred by ants and thus maintaining high pollinator visitation rates.

**Conclusions:**

Our findings indicate that reproduction-related traits in *R. ornata*, including those involved in pollinator attraction and reward and florivore defense, are highly effective and work in concert to maximize plant reproductive success. Therefore, a main risk that *R. ornata* faces is the decline or disappearance of hawk moths and other lepidopterans given its extreme specialization and high dependence on pollinators, and conservation efforts should include habitat protection for both *R. ornata* and its pollinators.

**Supplementary Information:**

The online version contains supplementary material available at 10.1186/s12862-024-02301-7.

## Background

Floral specialization reflects evolutionary diversification that is driven by selective pressures associated with reproduction, such as specific plant-pollinator interactions [1–4]. Concepts to classify floral traits (e.g., morphology, chemistry, phenology) that are linked to specific functional groups of pollinators are known as pollination syndromes [5–11]. These pollination syndromes allow researchers to test hypotheses about the predicted pollinators of specific plant species.

Although various plants, such as many orchid and cacti species, have exhibited a high degree of predictive power regarding pollination syndromes, with actual or potential pollinators matching predicted pollinators [12, 13], unexpected mismatches have been observed in other plant species [14, 15]. For example, species of *Castilleja* were identified to have bee, fly, and hummingbird pollination syndromes. However, none of these syndromes were found to be strongly predictive, as the plants received similar numbers of visits from other pollinator guilds [14]. Furthermore, the majority of plants possessing highly specialized flowers exhibit a facultative selfing strategy [8], in contrast with the common perception that floral specialization primarily serves to enhance out-crossing pollination [16]. Indeed, these specialist plants display a clear tendency for out-crossing, but autonomous selfing plays a crucial role in ensuring reproductive success under certain circumstances, such as when pollinators are scarce or absent [8, 17].

*Rivea ornata*, a rare species from the morning glory family, is known for its moth pollination syndrome, which is a feature distinctive to the genus [18, 19]. This plant species was found to influence interactions with other organisms via external secretory structures, such as the nectary disc where nectar is produced as a reward for pollinators [19]. Based on opportunistic observations, at least three different insect groups were considered potential pollinators of this plant species, i.e., skippers, hawk moths, and cockroaches [19]. However, empirical evidence is still needed to determine the actual pollinators of *R. ornata* (i.e., those that contribute significantly to reproductive success) given conflicts in the predictive validity of pollination syndromes reported for related species that share the moth pollination syndrome. For instance, pollination in the wide-spread *Ipomoea alba* was limited exclusively to hawk moths [20], whereas the island-inhabiting *I. habeliana* was visited by other insects in addition to hawk moths [21]. Moreover, the mating system of *R. ornata* remains unknown, precluding a comprehensive assessment of its specialized flowers and implications for plant reproductive success.

Antagonistic interactions were also preliminarily observed between *R. ornata* and its florivores [19]. This detrimental type of interaction is known to be another important determinant of reproductive success [22–24]. Although *R. ornata* was found to possess an indirect defense mechanism involving guard ants during early floral development, ants were noticeably absent on mature flowers, leaving them vulnerable to florivores such as katydids [19]. Reports examining florivory in *Daustinia montana* and *Ipomoea carnea* subsp. *fistulosa* revealed that damaged corollas, which are important elements for pollinator attraction, led to decreased pollinator visitation rates, resulting in a decline in plant reproductive success as a consequence [25, 26]. On the other hand, several plant species have evolved mechanisms to mitigate the negative effects of florivory, including changes in floral growth rate and apparency, production and allocation of chemical defenses, and the timing of flowering peaks [22, 27, 28]. However, the impact of corolla damage on pollination in *R. ornata* has never been evaluated, which hinders our understanding of how the species is disadvantaged or, conversely, how it copes to maximize reproductive fitness.

Therefore, this study aimed to (i) examine the biology of floral traits related to pollination, (ii) investigate the mating system of *R. ornata*, (iii) identify floral visitors and effective pollinators, and (iv) examine the effects of corolla damage on reproductive success. Gathering information on multiple aspects of floral biology and ecology is necessary for understanding how *R. ornata* balances pollinator attraction with florivore defense.

## Materials and methods

### Study species and study sites

*Rivea ornata* is a perennial shrub growing in the understory of deciduous dipterocarp forests and mixed forests [29]. Leaves are cordate with a pair of nectaries located at the petiole apex. Inflorescences are cymose, axillary and terminal, containing 4–5 flowers. Flowers are nocturnal, white, fragrant, and comprise five petals fused into a tube and limbs. The five stamens are included and epipetalous. The nectary disc is annular and encircles the base of the ovary. The two stigmas are oblong and included. The ovary contains four ovules. Fruits are dry and horizontally dehiscent [29].

*Rivea ornata* is found sparsely across the Indian subcontinent, extending through the Eastern Himalaya to Indochina, and is considered rare in Thailand. Data for this study were collected from the two largest known populations in Thailand, which allowed us to examine regional differences in pollinator visitation and reproductive success. The first population consisted of more than 30 individuals occupying around 17,000 m^2^ of pine-deciduous dipterocarp forest in a community reserve forest in Khun Yuam district, Mae Hong Son province in northern Thailand at an altitude of 590 m above sea level (a.s.l). The second population contained around 30 individuals occupying approximately 15,000 m^2^ of dry deciduous dipterocarp forest in a protected area (under the Plant Genetic Conservation Project under the Royal Initiative of Her Royal Highness Princess Maha Chakri Sirindhorn) in Phu Phan district, Sakon Nakhon province in northeastern Thailand at an altitude of ca. 300 m a.s.l. Plants were identified by Natthaphong Chitchak and voucher specimens (northern population: *NC & PT 45*,* 63*; northeastern population: *NC & PT 60*) were prepared following standard methods for plant taxonomy [30] and deposited at the Forest Herbarium in Bangkok, Thailand (BKF).

### Biology of floral traits related to pollination

The flowering and fruiting phenology of *R. ornata* was observed through field observations throughout the year. Data were recorded twice a month from 15 to 17 plant individuals per observation round throughout 12 consecutive months. Due to field constraints, observations were conducted only within the northern population with assistance from locals.

Flower longevity was observed through opportunistic field observations and video recordings, recording the times of flower opening and wilting, along with sunset and sunrise times.

Stigma receptivity was determined by peroxidase activity tested using a hydrogen peroxide solution [31]. Intact, virgin stigmas (still attached to styles) were collected at three time points, i.e., pre-anthesis (1600 h), anthesis (2200 h), and post-anthesis (0800 h), and were immediately submerged under 6% hydrogen peroxide. Submerged stigmas were observed for air bubbles and the number of bubbles floating up within a three-minute period were recorded. Five stigmas per time point were used from each population.

Pollen count and viability were assessed using pollen from undehisced anthers. Pollen grains were extracted and then dyed with either iodine solution or modified Alexander’s stain [32] (using 10 flowers per stain per population) to differentiate viable and non-viable pollen based on the stainability of starch and protoplasm, respectively. The stained pollen grains were observed and counted under light microscope (Olympus CX21) equipped with a Sony α6400 digital camera and visualized using ToupView software (ToupTek, China).

Nectar volume was quantified by measuring the length of nectar collected in a 7.5-cm capillary tube with a 60-µl capacity (Hirschmann, Germany). Nectar sugar concentration was measured by a handheld refractometer (Atago N1, 0–32%). Nectar volume and sugar concentration were examined from accumulated nectar collected at 0700 h (a single collection at the end of anthesis) and from nectar standing crop (repeated nectar removal throughout anthesis) collected at four time points, i.e., 1900, 2300, 0300, and 0700 h. Accumulated nectar was collected from 30 flowers from the northern population and 14 flowers from the northeastern population. Standing crop nectar was collected from nine flowers from the northern population only given field constraints and limited flower availability in the northeastern population. The quantity of nectar sugar per flower was calculated as the product of nectar volume and sugar concentration following [33].

### Mating system

To determine the mating system of *R. ornata*, a pollination experiment with five treatments was conducted: natural pollination (flowers were not manipulated), open pollination with emasculation (anthers were removed and then flowers were exposed to visitors as normal), hand cross-pollination (flowers were emasculated and received pollen from a different plant individual), hand self-pollination (flowers were pollinated using their own pollen), and autonomous self-pollination (flowers were enclosed in fine mesh bags to prevent animal visits). The hand cross-pollination and hand self-pollination treatments were also covered with fine mesh bags to prevent contamination of pollen from other sources. For treatments using emasculated flowers, undehisced anthers were removed from mature flower buds by making a small hole in the corolla tube wide enough to insert clean forcep tips to remove anthers. In the northern population, 308 flowers from 22 replicates were used during 14–16 September 2019 and 15–19 September 2020, and 63 flowers from seven replicates were used in the northeastern population during 7–11 September 2020. Each replicate was either a single plant individual or, when individual plants did not have enough flowers for all five treatments, a cluster of 2–4 individuals all within 1 m of each other that were likely siblings or clones. All replicates were at least five meters away from each other.

After the end of anthesis, all study flowers were covered with mesh bags to prevent fruit damage and loss. Study flowers were assessed for fruit development around six weeks later. Fruits and unfertilized ovaries were counted to quantify fruit set (i.e., fruit presence or absence). All fruits (with sepals and receptacles detached) were dried in a hot air oven (Binder FD240, Germany) at 60 °C for three days and then weighed using a digital balance (Aczet CY323, India). Afterwards, seeds were extracted from fruits in order to measure seed set (number of seeds per fruit) and seed weight.

### Floral visitation

Floral visitors were observed using video recordings and time-lapse photos taken every 2 s via action cameras (Yi Lite and SJCAM SJ4000X). Red light was used to enhance the visibility of flowers and floral visitors captured by cameras as this wavelength of color is almost imperceptible to most insect eyes and the least likely to disturb normal insect behavior [34]. Visitor observations were conducted for 8 nights in the northern population (14–16 September 2019 and 15–19 September 2020) and for 5 nights in the northeastern population (7–11 September 2020). Cameras were set to start recording shortly before floral anthesis (1700–1800 h) and collected footage until the following morning (0700–1100 h), covering the entire period from flower opening (1800–1900 h) to wilting (0430–1000 h). In total, 80 flowers from 36 inflorescences (1–4 blooming flowers per inflorescence) were observed (50 flowers from 24 inflorescences in the northern population and 30 flowers from 12 inflorescences in the northeastern population). Due to the relatively low resolution of video footage and photos, visitors were classified into one of nine groups: (i) nocturnal and (ii) diurnal hawk moths (Sphingidae, Lepidoptera, which hover when feeding from flowers), (iii) settling moths (moths in families other than Sphingidae which land and sit on flowers while feeding), (iv) skipper butterflies (Hesperiidae, Lepidoptera), (v) katydids (Tettigoniidae, Orthoptera), (vi) beetles (Coleoptera), (vii) cockroaches (Blattodea), (viii) crickets (Grylloidea, Orthoptera), and (ix) unknown visitors. The potential roles of these visitors were determined based on their interactions with flowers; animals that consumed floral tissue were considered florivores and animals that contacted the corolla throat (where stigmas and anthers are located) and/or were observed carrying pollen were considered potential pollinators. No visitors were ever observed exhibiting both types of behaviors.

### Pollination efficiency of flying visitors

To examine the pollination contribution of visitors that approach *R. ornata* corollas by flying (hawk moths, settling moths, and skippers) in comparison to other floral visitors, lepidopteran exclusion experiments were performed. Clear plastic sheets (21 × 21 cm) were placed approximately 10 cm in front of the flowers (parallel to the face of the flower). The plastic prevented visits from lepidopterans, which always approach flowers during flight from the front (i.e., the adaxial side of the corolla; Additional file 1: Video S1), meaning that only visitors that approached flowers by crawling (e.g., cockroaches and crickets; Additional file 1: Video S1) were able to access flowers. The plastic sheets were set before floral anthesis (1600–1800 h) and left throughout the night. Unmanipulated flowers were used as the control group. In the northern population, 74 flowers from 13 replicates were examined (during 15–19 September 2020 and 17–22 September 2022), and 24 flowers from 10 replicates were examined in the northeastern population (during 7–11 September 2020 and 12–15 September 2022).

### Effect of floral damage on reproductive success

To test whether corolla damage affects natural fruit and seed set, we simulated artificial florivory using scissors and also examined the effects of natural florivory. Preliminary observations revealed that insects typically consume the delicate areas of the corolla limbs between the midpetaline bands (i.e., the plicae), while avoiding the thicker midpetaline bands (Additional file 1: Video S1). We therefore used two levels of artificial florivory that mimicked the damage patterns observed in the field, and a third level that was more extreme. Specifically, for medium-level artificial damage, we cut out the corolla tissue between 2 of the 5 midpetaline bands. For severe artifical damage, we cut off all of the delicate corolla tissue, leaving only the midpetaline bands and corolla tube. For total artifical damage, we cut off all corolla limbs (including midpetaline bands), leaving only the corolla tube. Artificial damage was inflicted as soon as flowers started to open and before pollinator visitation. Unmanipulated, undamaged flowers were used as the control group. On the night of anthesis, if the medium-level artificially damaged flowers were consumed by natural florivores, resulting in at least 60% damage to the total corolla limb area, they were reassigned to the severe-level artificially damaged flowers (*n* = 3 flowers). Only intact flowers or those receiving minor damage (not more than 20% of the corolla limb destroyed by natural florivores) were included in the control group. Naturally damaged flowers were also included in the analysis under two levels: medium-level natural damage (21–60% of delicate corolla limb tissue eaten) and severe-level natural damage (61–100% of delicate corolla limb tissue eaten). Total corolla limb damage (including to midpetaline bands) was not found naturally. We used 99 flowers from 16 replicates in the northern population, and 40 flowers from seven replicates in the northeastern population. Floral damage experiments were conducted in 2022 during the same study period as the lepidopteran exclusion experiments.

### Statistical analyses

R version 4.3.1 [35] was used in all analyses. For the pollination experiment, generalized linear mixed modeling (GLMM; function *glmer* in package “lme4”) was performed for fruit set (using a binomial distribution) and seed set (using a Poisson distribution) and linear mixed modeling (LMM; function *lmer* in package “lme4”) [36] was performed for fruit and seed weight to assess differences among pollination treatments and populations. Treatment and study site (i.e., population) were treated as fixed factors, and plant replicate was treated as a random factor. Similarly, GLMM with a Poisson distribution was used to test whether the number of floral visits differed by insect group. Visitor group and study site were treated as fixed factors while flower ID was treated as a random factor. Likelihood ratio tests were used to examine the significance of fixed factors and, for significant factors, Tukey’s tests were used for post hoc analyses (function *emmeans* in package “emmeans”, visualized with function *cld* in package “multcomp”) [37]. Moreover, visitation rates throughout anthesis (starting from 1800 h, which was the first hour that all flowers were open) were fitted via linear modelling (function *lm* in package “stats”) to assess the trend in visitation over time. Finally, permutational analysis of variance (PERMANOVA) was conducted to compare floral visitor composition between study sites using Bray-Curtis dissimilarity with 999 permutations (function *adonis2* in package “vegan”) [38]. For experiments or observations that were conducted over two years, data from both years were pooled given small sample sizes and low interannual variance. Numerical results are presented throughout the text as mean ± standard error (SE).

## Results

### Biology of floral traits related to pollination

Growth stages in the annual life cycle of *R. ornata* were categorized into ten stages (Additional file 2: Fig. S1). Flower bud formation was prominent in August and peak blooming occurred during September. Fruit development became apparent in late September through November, followed by fruit drying and dehiscence in December.

Flowers of *R. ornata* opened around 1815 h ± 11 min, shortly before sunset (1846 h ± 8 min), and began wilting around 0709 h ± 16 min, around an hour after sunrise (0600 h ± 7 min). The average duration of floral longevity was 11.5 ± 1.1 h.

Stigma receptivity as assessed by peroxidase activity (generating oxygen bubbles through the breakdown of hydrogen peroxide), was observed to be greater during anthesis (with up to two bubbles floating to the surface within a three-minute interval) compared to both pre-anthesis and post-anthesis stages (where only air bubbles clinging to the stigmas were observed).

Anthers were found to produce 3,112 ± 277 pollen grains per flower. Pollen viability, measured using modified Alexander’s stain and iodine solution, was 98.27 ± 0.44% and 98.84 ± 0.07%, respectively.

**Fig. 1:**
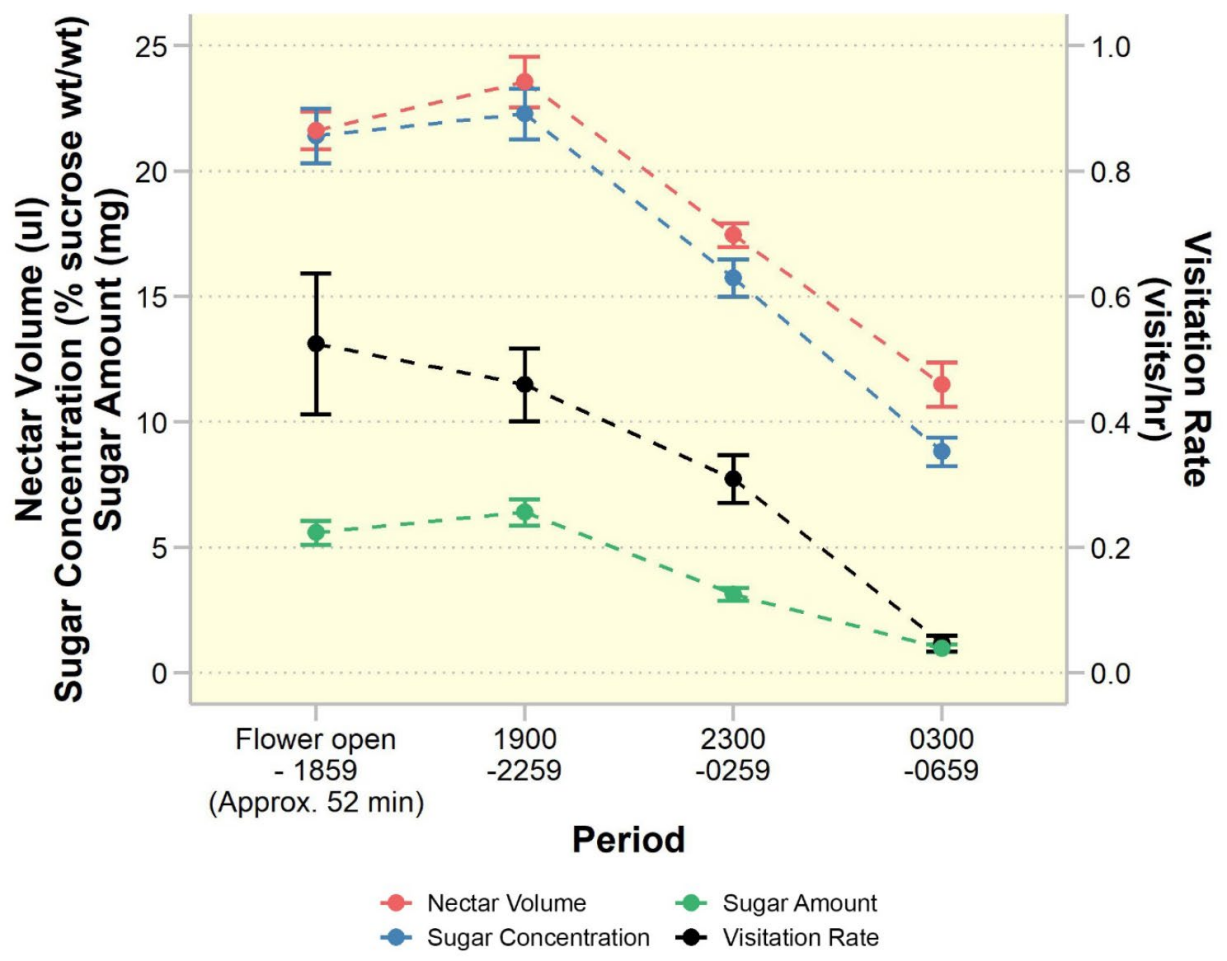
Floral nectar production of *Rivea ornata* and hawk moth visitation rates to flowers at four time periods during anthesis. Study variables related to nectar production (collected from *n* = 9 flowers), i.e., nectar volume (red), sugar concentration (blue), and sugar amount (green), are plotted against visitation rate (black) (observed from *n* = 80 flowers). Scale on the left y-axis corresponds to nectar production variables. Scale on the right y-axis is used for visitation rate. Dots and error bars denote means and standard errors

Without nectar removal during anthesis, each individual flower yielded 44.25 ± 1.83 µl of nectar with a sugar concentration of 19.93 ± 0.13% sucrose (wt/wt), equivalent to a total sucrose content of 10.12 ± 0.44 mg. Nectar production varied throughout anthesis, with the highest production observed between 1900 and 2300 h, followed by a subsequent decline (Fig. 1). Furthermore, the total volume of nectar from flowers subjected to nectar removal every four hours was greater (73.33 ± 1.85 µl) compared to those without regular nectar removal, whereas average sugar concentration (16.88 ± 1.04% sucrose (wt/wt)) showed minimal variation between the two methods of nectar removal.

### Mating system

**Fig. 2:**
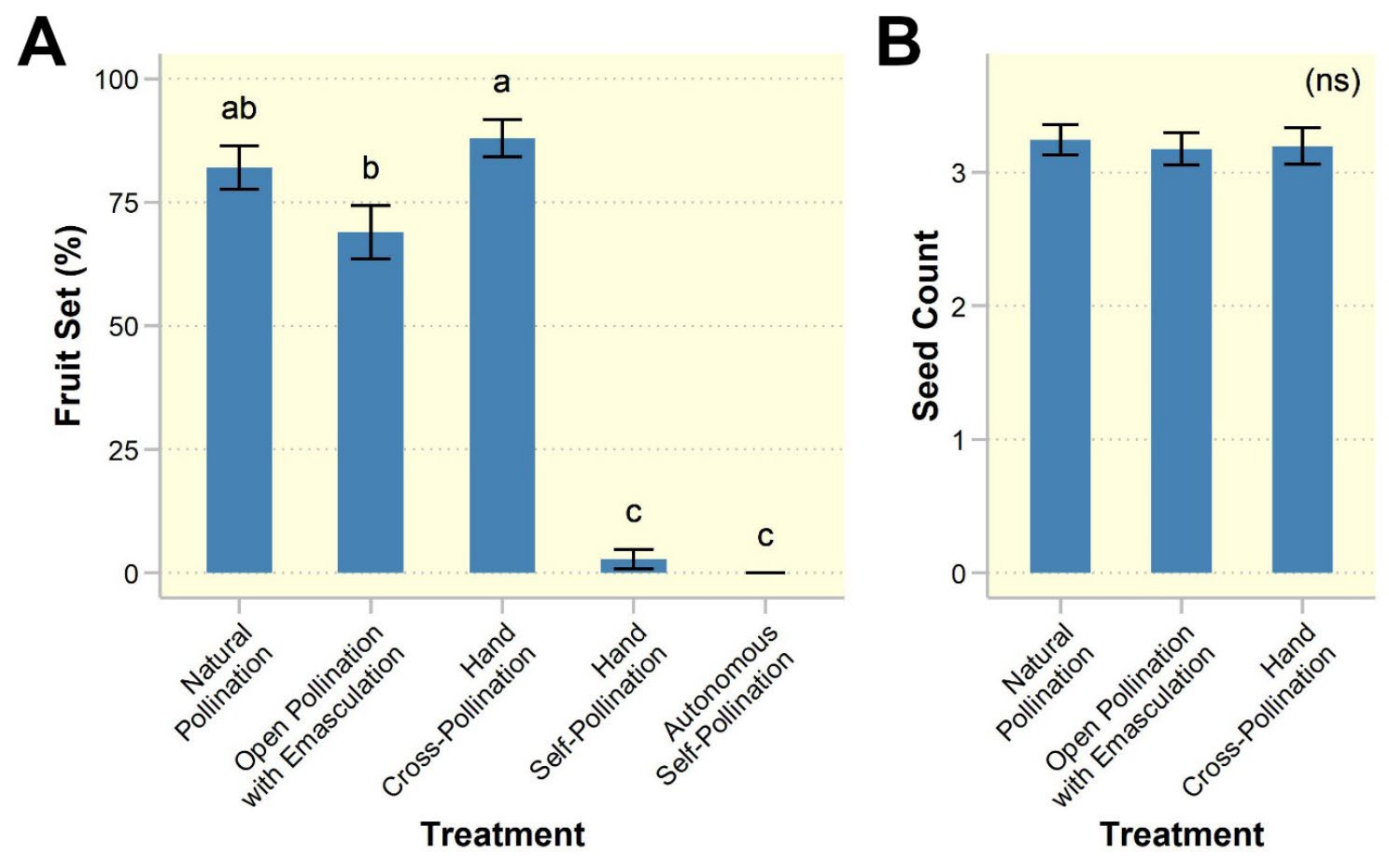
Reproductive success of *Rivea ornata* evaluated by pollination experiments. (**A**) Percentage fruit set for each of the five treatments; (**B**) Seed count (seeds per fruit) from the three treatments that successfully set fruit. Bars and error bars denote means and standard errors, *n* = 29 plants per treatment. Treatments with different letters are significantly different (*p* < 0.05). The results of fruit and seed weight are shown in Additional file 2: Fig. S2. Abbreviation: ns, not significant

Analysis revealed that pollination treatment ($\:{X}_{3}^{2}$ = 267.98, *p* < 0.001), but not study site ($\:{X}_{1}^{2}$ = 0.65, *p* = 0.42), had a significant effect on fruit set (Fig. 2A). The autonomous self-pollination treatment did not yield any fruit. Post hoc analysis showed that the percentages of fruit set in the natural pollination (82.05 ± 4.37%), open pollination with emasculation (68.99 ± 5.42%), and hand cross-pollination (88.00 ± 3.78%) treatments were significantly higher than those in the hand self-pollination (2.82 ± 1.98%) and autonomous self-pollination (0%) treatments (*p* < 0.001; Fig. 2A). Fruit set from open pollination with emasculation treatment was significantly lower than that of the hand cross-pollination treatment (*p* = 0.03), but not the natural pollination treatment (*p* = 0.25). Among the three treatments that set fruit, there were no significant differences in dry fruit weight ($\:{X}_{2}^{2}$ = 1.98, *p* = 0.37; Additional file 2: Fig. S2A), seed set ($\:{X}_{2}^{2}$ = 0.05, *p* = 0.98; Fig. 2B), or seed weight ($\:{X}_{2}^{2}$ = 0.21, *p* = 0.90; Additional file 2: Fig. S2B).

### Floral visitation

**Fig. 3:**
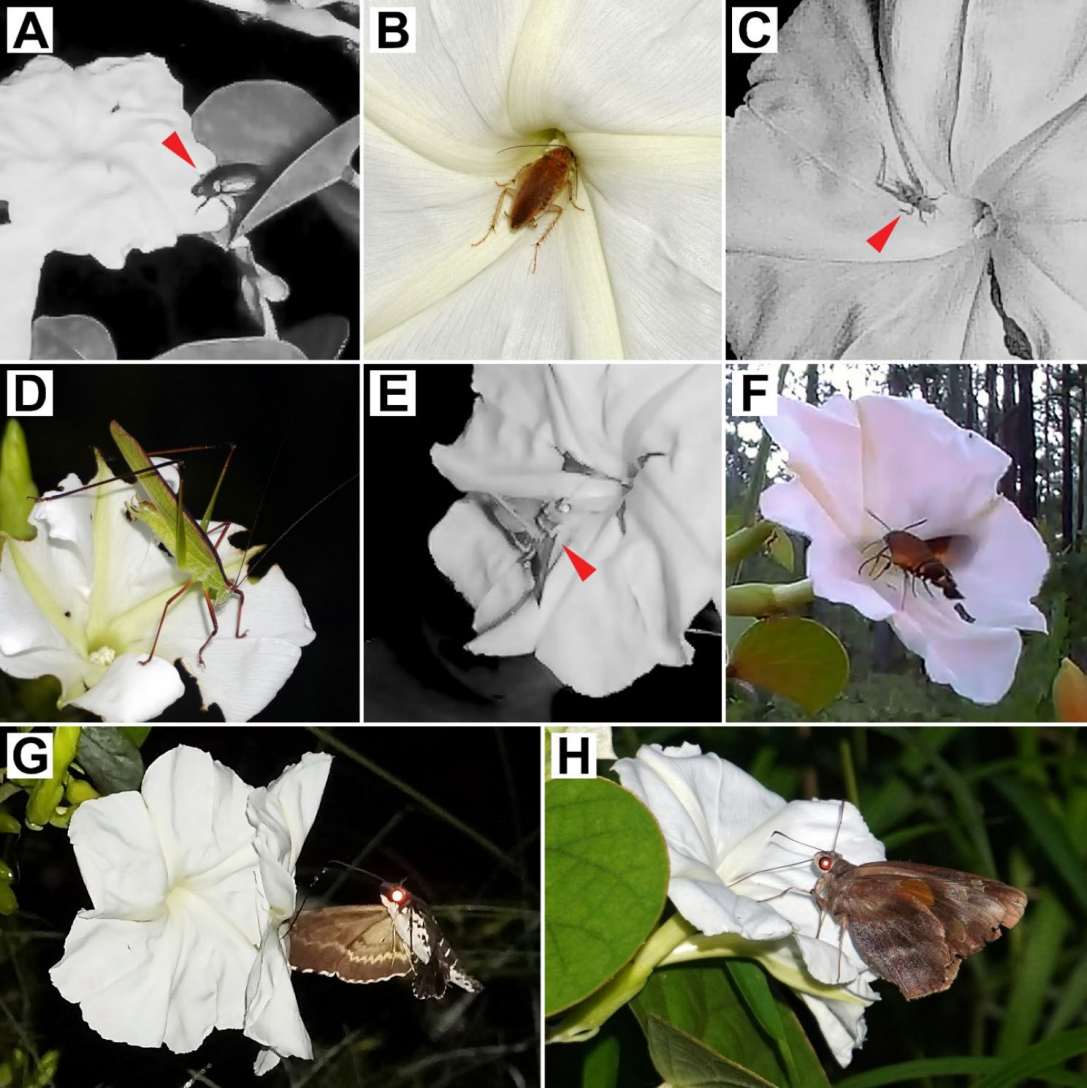
Floral visitors of *Rivea ornata*. (**A**) Beetle; (**B**) Cockroach; (**C**) Cricket; (**D**) Katydid; (**E**) Settling moth; (**F**) Diurnal hawk moth; (**G**) Nocturnal hawk moth; (**H**) Skipper. Arrowheads in greyscale panels (**A**, **C**, **E**) locate floral visitors

Across a total of 530 observation hours, 560 interactions were recorded. Animal visitors were observed at the large majority of flowers (97.5%), and only two out of 80 observed flowers were not visited at all. Examining the behavior of visitors at *R. ornata* flowers revealed that katydids and beetles are florivores (accounting for 98 interactions combined), while other visitors were found to be potential pollinators (accounting for 462 interactions). Florivores were observed consuming the delicate tissue of the corolla limbs but left the midpetaline bands, corolla tube, and reproductive organs (pistils and stamens) undisturbed (Fig. 3; Additional file 1: Video S1).

**Fig. 4:**
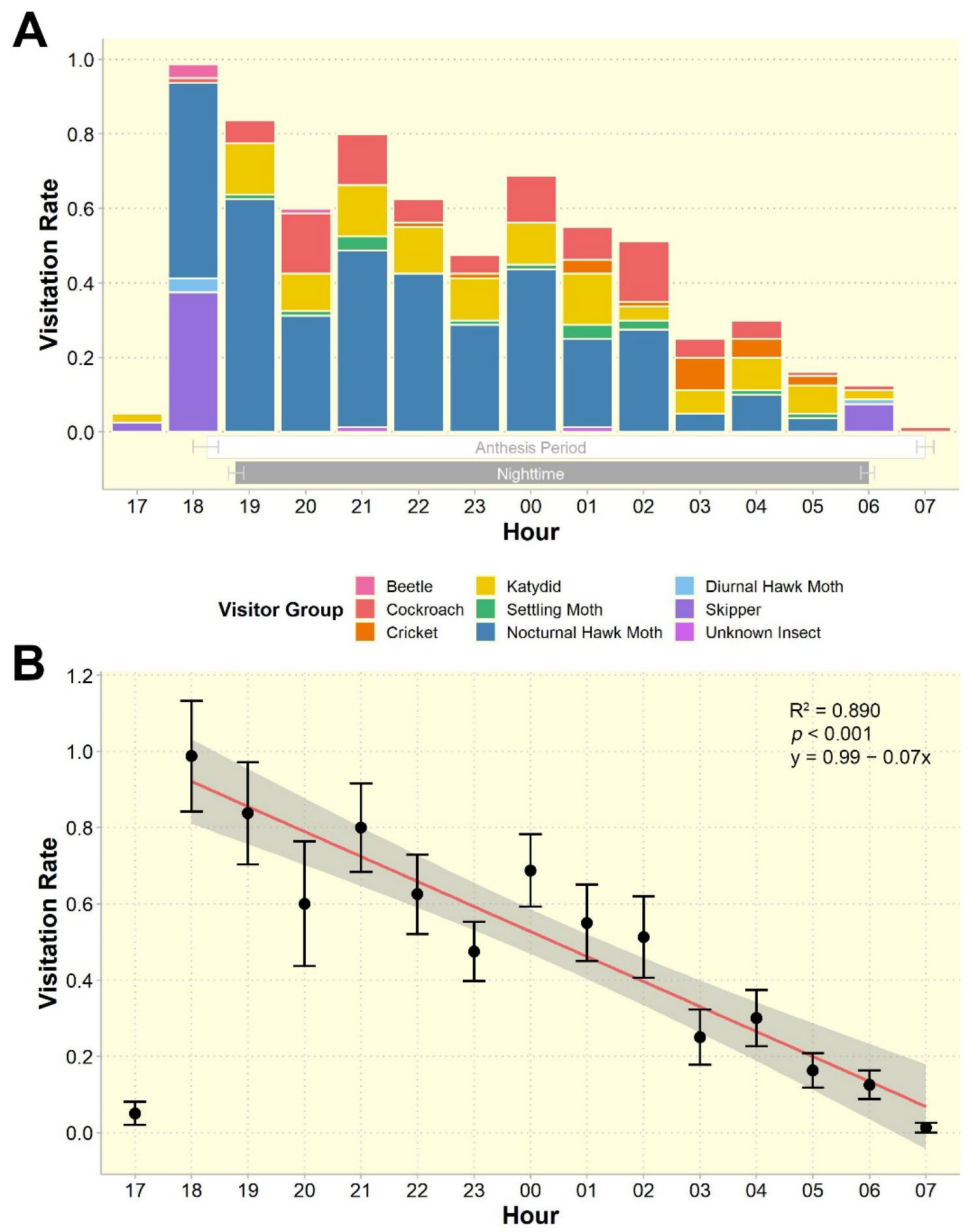
Visitation rates (visits per flower per hour) of *Rivea ornata* floral visitors throughout anthesis. (**A**) Mean visitation rates showing the proportion of visits contributed by different visitor groups (represented by different colors). White and grey horizontal rectangles, with error bars, indicate periods of anthesis and nighttime, respectively; (**B**) Visitation rates fitted via linear modelling. Dots and error bars denote means and standard errors of visitation from all visitors. Regression line is shown in red and the 95% confidence interval is shown by the grey shaded region. Note that the model included visits from 1800 h onwards as it was the first hour that all flowers were open, *n* = 530 observation hours at 80 flowers

Almost all visitors were nocturnal and only diurnal hawk moths and skippers were active during the day (Fig. 3). Overall visitation rate was found to differ significantly throughout anthesis (*F*_1,12_= 97.29, *p* < 0.001; Fig. 4). Visitation was highest during 1800–1900 h (0.99 ± 0.15 visits/flower), shortly after flowers opened, and exhibited a gradual decline throughout the rest of anthesis (Fig. 4).

**Fig. 5:**
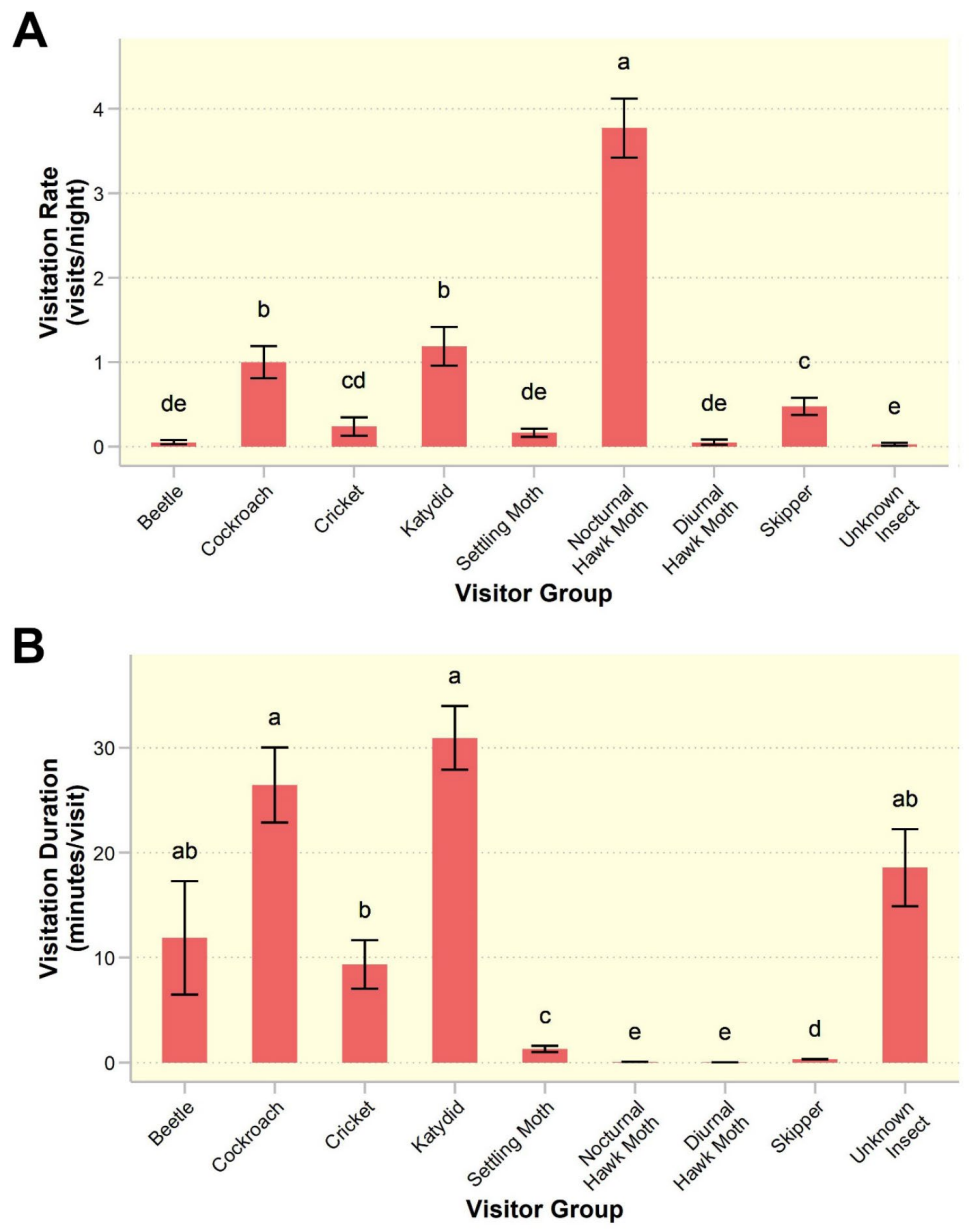
Patterns of activity at *Rivea ornata* flowers for each visitor group. (**A**) Visitation rate per night; (**B**) Visit duration per visit. Bars and error bars denote means and standard errors, *n* = 530 observation hours at 80 flowers. Visitor groups with different letters are significantly different (*p* < 0.05)

Visitation rate also differed significantly among visitor groups ($\:{X}_{8}^{2}$ = 899.82, *p* < 0.001; Fig. 5A), but not between study sites ($\:{X}_{1}^{2}$ = 2.63, *p* = 0.10). Turkey’s post hoc results revealed that nocturnal hawk moths visited significantly more often than all other visitor groups (3.78 ± 0.35 visits/flower/night) (*p* < 0.001; Fig. 5A). Moreover, visit duration was found to be significantly different among visitor groups ($\:{X}_{8}^{2}$= 1347.4, *p* < 0.001; Fig. 5B). The lepidopteran visitors (hawk moths, settling moths and skippers), spent significantly less time per visit than other visitor groups (Fig. 5B).

The composition of floral visitors was found to be significantly different between the two study sites (*F*_1,33_= 11.64, *p* < 0.001; Additional file 2: Fig. S3). While nocturnal hawk moths and skippers were the primary visitors observed at both study sites, the relatively higher visit frequencies of katydids in the northeastern population and that of diurnal hawk moths and cockroaches in the northern population separated the two populations (Additional file 2: Fig. S3).

### Pollination efficiency of lepidopterans versus other floral visitors

**Fig. 6:**
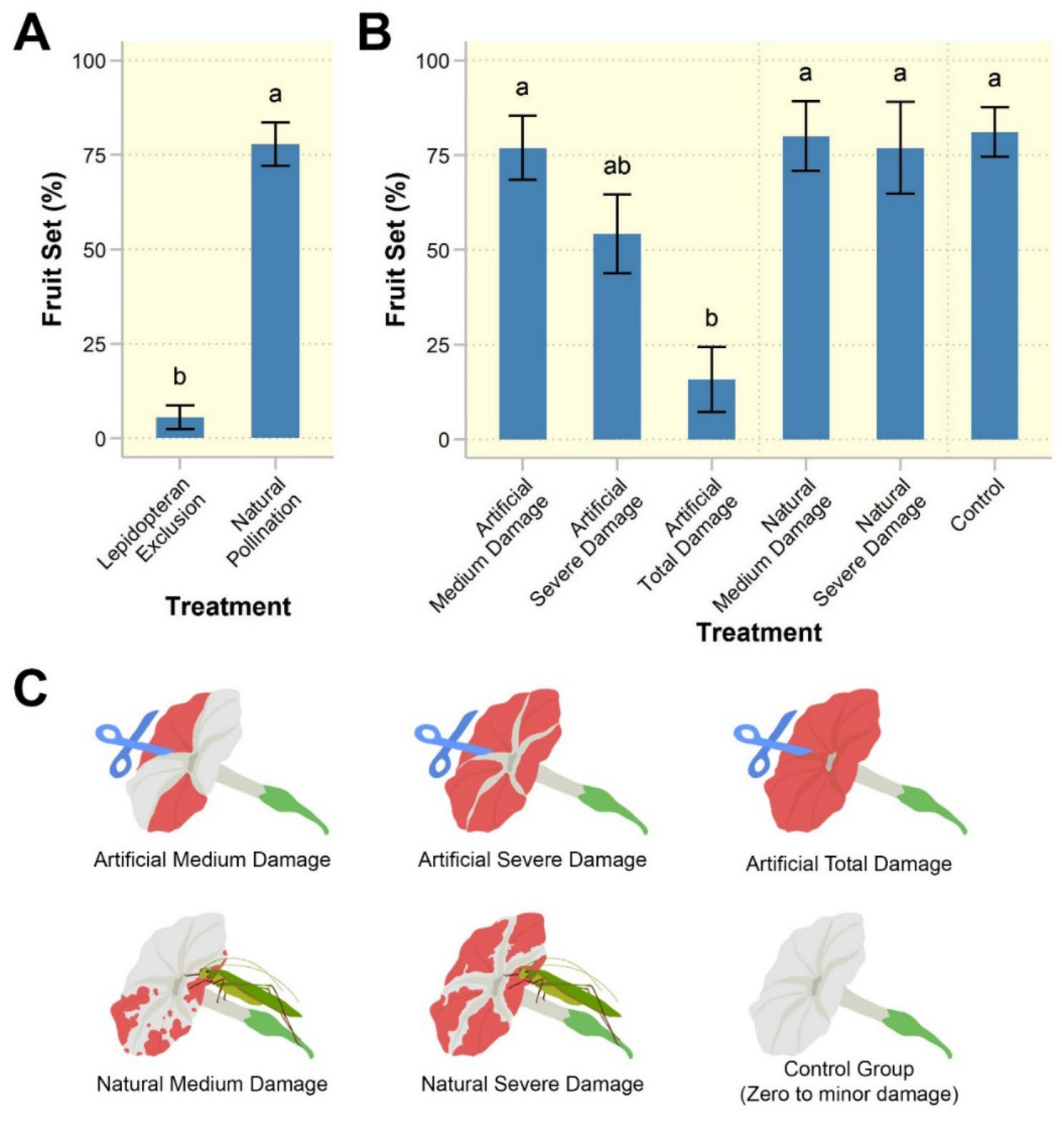
Results of experiments examining the effective pollinators of *Rivea ornata* and the impact of florivory on reproductive success. (**A**) Percentage of fruit set when lepidopterans were excluded versus when they were allowed to visit (natural pollination); (**B**) Percentage of fruit set in the field under different levels of floral damage induced either artificially (via scissors) or naturally (via florivores): control (0–20% of delicate corolla limb tissue removed), medium damage (21–60%), severe damage (61–100%), and total damage (all corolla limbs including midpetaline bands cut off). Note that total damage was never observed naturally in the wild. (**C**) Drawings illustrating the levels of floral damage in the florivory experiment (red indicates areas removed by scissors or by florivore consumption). Bars and error bars denote means and standard errors, *n* = 23 plants per treatment. Treatments with different letters are significantly different (*p* < 0.05)

The exclusion of lepidopterans significantly reduced fruit set ($\:{X}_{1}^{2}$ = 66.43, *p* < 0.001; Fig. 6A). The percentage of fruit set in the lepidopteran exclusion treatment (5.56 ± 3.15%) was significantly lower than the control group (natural pollination by all visitors, including lepidopterans) (77.78 ± 5.71%) (*p* < 0.001; Fig. 6A).

### Effect of floral damage on reproductive success

Floral damage ($\:{X}_{5}^{2}$ = 30.15, *p* < 0.001; Fig. 6B), but not study site ($\:{X}_{1}^{2}$ = 1.02, *p* = 0.31), significantly affected fruit set. Post hoc results showed that artificial damage to the entire corolla limb resulted in the lowest fruit set and was significantly different from the control group (minor to zero damage) and all other treatments (*p* < 0.05; Fig. 6B) except for the artificial-severe damage treatment (*p* = 0.14; Fig. 6B). The fruit set of flowers incurring natural damage (at both medium and severe levels) was not significantly different from that of the control group, nor was the fruit set of the artificially damaged flowers at the medium and severe levels (*p* > 0.05; Fig. 6B).

## Discussion

### Mating system and pollination syndrome of *Rivea ornata*

Our pollination experiments confirmed that *R. ornata* is completely self-incompatible and obligately xenogamous, indicating that the species is completely dependent on pollinators. The high consistency of plant reproductive success within each treatment, in spite of the fact that replicates consisted of different numbers of plants and plants bore different numbers of flowers, strongly supports self-incompatibility rather than differential resource allocation [39, 40]. In the Convolvulaceae, mating systems are diverse. Self-incompatibility has been reported in several species across various genera, e.g., *Argyreia siamensis* [41], *Distimake palmeri* [42], *Ipomoea marcellia* [43], *Stictocardia tiliifolia* [44], and *Calystegia soldanella* [45]. Other species are self-compatible but still tend to favor outcrossing (facultative xenogamy), e.g., *I. aquatica* [46], *D. macrocalyx* [47], and *Jacquemontia sandwicensis* [48], although a few species have been found to favor selfing over outcrossing (facultative autogamy), e.g., *J. multiflora* [49], *Xenostegia tridentata* [50], and *Cuscuta lupuliformis* [51].

Our results also confirm that *R. ornata* is pollinated by hawkmoths (Sphingidae, Lepidoptera), as predicted by its floral traits. This pollination syndrome is relatively uncommon within the Convolvulaceae, with most species having diurnal and generalist flowers that are typically visited by bees, and sometimes by flies and butterflies [48, 52, 53] or wasps [54]. Pollinator specialization in this family is usually found in the form of ornithophily (bird pollination), especially in neotropical *Ipomoea* [20]. Other pollination syndromes, such as moth and bat syndromes, are less common. The mating systems of these specialized plants are diverse, with some exhibiting self-compatibility, e.g., the bird-pollinated *I. hederifolia* and the moth-pollinated *I. alba* [20], and others exhibiting self-incompatibility, i.e., the bat-pollinated *I. marcellia* and the moth-pollinated *Distimake palmeri* [42, 43]. Moreover, a recent study examining the self-incompatible *Argyreia mekongensis* and *A. versicolor* found that these Asiatic species exhibit a carpenter bee pollination syndrome, also uncommon in the family, as evidenced by floral morphology and precise pollen placement [55]. The predicted pollinators of most of these specialist species were highly accurate in matching their actual floral visitors, similar to our findings for *R. ornata* in this study. The congruence between peak nectar production and peak hawk moth activity (Fig. 1) [42, 56] provides further support that *R. ornata* is sphingophilous.

The decline in pollinator activity throughout anthesis may be due to multiple factors. A likely reason is that nocturnal pollinators often exhibit the highest foraging activity early in the evening, when night-blooming flowers first open and nectar production is greatest [57, 58]. Indeed, in *R. ornata* flowers, both nectar quantity and sugar concentration decline steadily throughout the night (Fig. 1), making later visits less profitable to pollinators than early visits. The decline in pollinator visitation may also be due to changes in floral scent that occur following fertilization, as has been demonstrated in many plant species [59 and references therein]. Such changes in floral cues are hypothesized to benefit both plants (e.g., by allowing them to conserve resources or reduce notice by florivores) and pollinators (e.g., by allowing them to discriminate between high-rewarding virgin flowers versus depleted flowers) [59], but further research is necessary to determine whether *R. ornata* uses such signaling.

Interestingly, *R. ornata* flowers are highly specialized in terms of pollinators, but they appear to lack reproductive assurance mechanisms, such as delayed selfing or attracting secondary pollinator groups. While specialized flowers are known to promote outcrossing, many species are also self-compatible and capable of reproducing via autonomous selfing, which serves as a backup strategy to ensure reproductive success when pollinators are scarce or absent [8, 17]. Moreover, specialist species that are self-incompatible often utilize other methods to ensure reproduction, such as by attracting floral visitors beyond those predicted by their pollination syndromes. For instance, the bat flowers *Ipomoea vespertilia* and *I. neei* employ a bet-hedging strategy by remaining open and producing nectar throughout the night until the following morning, mitigating the risk of insufficient bat visitation by compensating with hummingbird visitation [20, 43]. However, our data indicate that this strategy is unlikely to be used by *R. ornata*. Despite the presence of other floral visitors, such as cockroaches (Blattodea) and crickets (Grylloidea, Orthoptera), such visitors are likely opportunistic and ineffective pollinators since our lepidopteran exclusion experiment proved that they set very little fruit. While non-lepidopteran insects do contact the stigmas and anthers located at the corolla tube entrance, we predict that they transfer relatively few pollen grains between different plants given that they spend up to 30 min on a single flower (possibly foraging on pollen and stigma exudates), and most of their movements may occur between flowers on the same plant given their crawling behavior (Additional file 1: Video S1).

While *R. ornata* flowers are primarily nocturnal, their anthesis period does overlap slightly with the activity of some diurnal insects, particularly diurnal hawk moths and skippers. These diurnal lepidopterans may actually be as effective in pollinating *R. ornata* flowers as nocturnal hawk moths, however, their visitation rates were found to be significantly lower than that of nocturnal hawk moths. We therefore predict that diurnal lepidopterans contribute less to *R. ornata* pollination than nocturnal hawk moths, although additional experiments are needed to test this prediction. Additionally, the opening of *R. ornata* flowers half an hour before sunset may not reflect selection to increase diurnal pollinator visitation, but may rather result from environmental cues. It was found that in *Ipomoea* species, flower opening is mainly controlled by hormonal regulation and environmental factors such as the dark/light cycle, humidity, and temperature [60, 61].

Extreme specialization and dependency on a single pollinator group is uncommon [62], but not unheard of. Similar findings of plants dependent on a single effective pollinator group have been reported in two other species exhibiting moth pollination syndromes, i.e., *I. alba* and *Distimake palmeri* [29, 42]. Our finding indicates that cross-pollination in *R. ornata* is primarily facilitated by nocturnal hawk moths, as they were the most frequently observed visitor group and their absence negatively affected fruit set. Thus, this plant-pollinator mutualism is necessary for *R. ornata* to reproduce in their natural habitats.

### Effect of florivory on *rivea ornata* reproduction

Our experiments unexpectedly revealed that natural florivory did not significantly affect fruit set in *R. ornata*. In contrast to our results, studies examining *Daustinia montana* and *Ipomoea carnea* subsp. *fistulosa* found that floral damage reduced plant reproductive success as damaged flowers were less attractive to pollinators [25, 26]. Indeed, florivory is generally thought to negatively impact plant fitness, either directly through the consumption of reproductive structures, i.e., stamens and pistils [26], or indirectly via the alteration or destruction of pollinator attractants and rewards, leading to reduced pollinator visitation and diminished reproductive success [63–65].

Katydids (Tettigoniidae, Orthoptera) were confirmed in this study to be the main florivores of *R. ornata*, damaging 77% of flowers in the northeastern population (23 out of 30 observed flowers) and 14% of flowers in the northern population (7 out of 50 observed flowers). The high fruit set found in our study populations, even following severe floral damage, may be explained by several factors. One reason may be that pollination is completed before or during the early stages of floral destruction, before severe damage reduces pollinator attraction. In the case of the moth flower *Distimake palmeri*, a single visit was found to be sufficient for successful fertilization [42]. This may also be the case for *R. ornata*, as almost all flowers received at least one visit by a hawk moth. Hawk moths were found to be capable of carrying pollen from plants in this family in quantities ranging from 12 to more than 300 pollen grains [42, 66], and a single *R. ornata* flower requires only four fully functioning pollen grains to fertilize the four ovules. Therefore, flowers of *R. ornata* may be successfully pollinated at the beginning of anthesis when pollinator visitation is highest (Figs. 1 and 4). Further supporting this hypothesis, katydid presence on *R. ornata* flowers was lowest when flowers first opened, but then remained fairly constant throughout the night (Fig. 4), indicating that floral damage increases gradually over time. Moreover, katydid presence on flowers did not deter hawk moth visits.

Another explanation may be that natural floral damage does not hinder pollinator attraction or the ability of pollinators to locate flowers. Our artificial damage treatments revealed that even when all the delicate tissue of corolla limbs were cut off, leaving only the corolla tube and midpetaline bands, they were still able to attract pollinators and set fruit, but when the midpetaline bands were also removed, fruit set was significantly reduced. These results indicate that the midpetaline bands are an important component of floral attractiveness and/or in helping pollinators locate flowers, likely in addition to olfactory cues produced by the glandular staminal trichomes of *R. ornata*, such as terpenes [19, 67]. Interestingly, katydids only consumed the delicate corolla limb tissue and not the midpetaline bands. Our additional anatomical examination of *R. ornata* flowers revealed that large laticifers are located within the midpetaline bands, but not in the thin, delicate areas of the corolla limb (Additional file 2: Fig. S4). Latex produced by laticifers in convolvulaceous plants is known for its role in defense against herbivores [68, 69], suggesting that it might be unpalatable to katydids, which could explain why they only consumed the delicate areas of the corolla limb. This defense strategy exhibited by *R. ornata* aligns with the optimal defense hypothesis, which predicts that plants allocate more defense to tissues that strongly affect plant fitness [22, 27, 28].

*Rivea ornata* also relies on guard ants for herbivore defense [19] and appears to have achieved a balance in using these ants to reduce florivory without deterring pollinators. Extrafloral nectaries are common in the Convolvulaceae, and secrete nectar to attract ants to vegetative parts via petiolar nectaries and to reproductive parts via receptacular nectaries [70–73]. Interestingly, *R. ornata* has not only petiolar nectaries on its leaves, but also calycinal glands on its flowers, an analogous structure that functions in attracting ants similar to receptacular nectaries [19]. In this study, it was confirmed that ants exclusively patrolled the flower buds. However, once the flowers are fully open, their patrolling remains confined to the calyx, where they forage on fluids secreted at the calycinal glands, leaving the corolla unguarded and vulnerable to florivory. While guard ants are generally beneficial to plants, their presence on flowers typically has a negative effect on reproduction, as they deter pollinators, resulting in a decrease in pollinator visitation rates [25, 74–76]. Therefore, our results suggest that *R. ornata* maximizes fitness by employing trade-offs, attracting guard ants but not on flowers where they would deter pollinators [77], and while the lack of patrolling on flowers results in some florivory, such damage is restricted to non-vital floral organs.

## Conclusions

*Rivea ornata* is a rare plant species, but this study found the species to be very fertile with high reproductive output. As *R. ornata* is self-incompatible, the high natural fruit and seed set and lack of pollen limitation indicate that its pollinators are effective and reliable. Furthermore, the effective pollinators (mostly nocturnal lepidopterans) were found to be consistent with its predicted pollination syndrome. *Rivea ornata* also exhibits strategies that effectively cope with the adverse effects of florivory, allowing them to maintain high reproductive success, which can be explained by the optimal defense hypothesis and functional trade-offs. Overall, our study indicates that reproduction-related traits in *R. ornata*, including those involved in pollinator attraction and reward and florivore defense, are highly effective and work in concert to maximize plant reproductive success.

Additionally, our findings suggest that the rarity of *R. ornata* is likely caused by factors unrelated to reproduction, such as habitat loss. This plant species typically inhabits open spaces, such as dry dipterocarp forests and forest edges, where it is susceptible to disturbance from human activity or grazing. Thus conservation plans for *R. ornata* should include protecting key habitats. Indeed, our study localities (the two largest natural populations in the country) were both located in forest reserves. Given that the mating system of *R. ornata* is obligate outcrossing and fully dependent on pollinators, and the finding that nocturnal hawk moths are the primary effective pollinators, the continued persistence of *R. ornata* in natural habitats relies tremendously on the co-occurence of its pollinators. Therefore, parallel conservation efforts must also focus on preserving populations of nocturnal hawk moths.

### Electronic supplementary material

Below is the link to the electronic supplementary material.

Supplementary Material 1

### Electronic supplementary material

Below is the link to the electronic supplementary material.

Supplementary Material 2

## Data Availability

The raw data supporting the conclusions of this article are available in the Mendeley Data repository, DOI: 10.17632/2n4vgpvzgs.1, (https://data.mendeley.com/datasets/2n4vgpvzgs/1).
