# Supplementary material for: Trades-offs between pollinator attraction and florivore defense maximize reproductive success in the self-incompatible Rivea ornata (Convolvulaceae)
Source: BMC Ecol Evol. 2024 Aug 30;24:115. doi: 10.1186/s12862-024-02301-7 (PMC11363511; doi:10.1186/s12862-024-02301-7)
Supplement: Supplementary file 2 — Supplementary Material 2 [file 12862_2024_2301_MOESM2_ESM.pdf]

**Additional file 2**

**Trades-offs between pollinator attraction and florivore defense maximize reproductive success in the self-incompatible *Rivea ornata* (Convolvulaceae)**

Natthaphong Chitchak, Alyssa B. Stewart, Paweena Traiperm

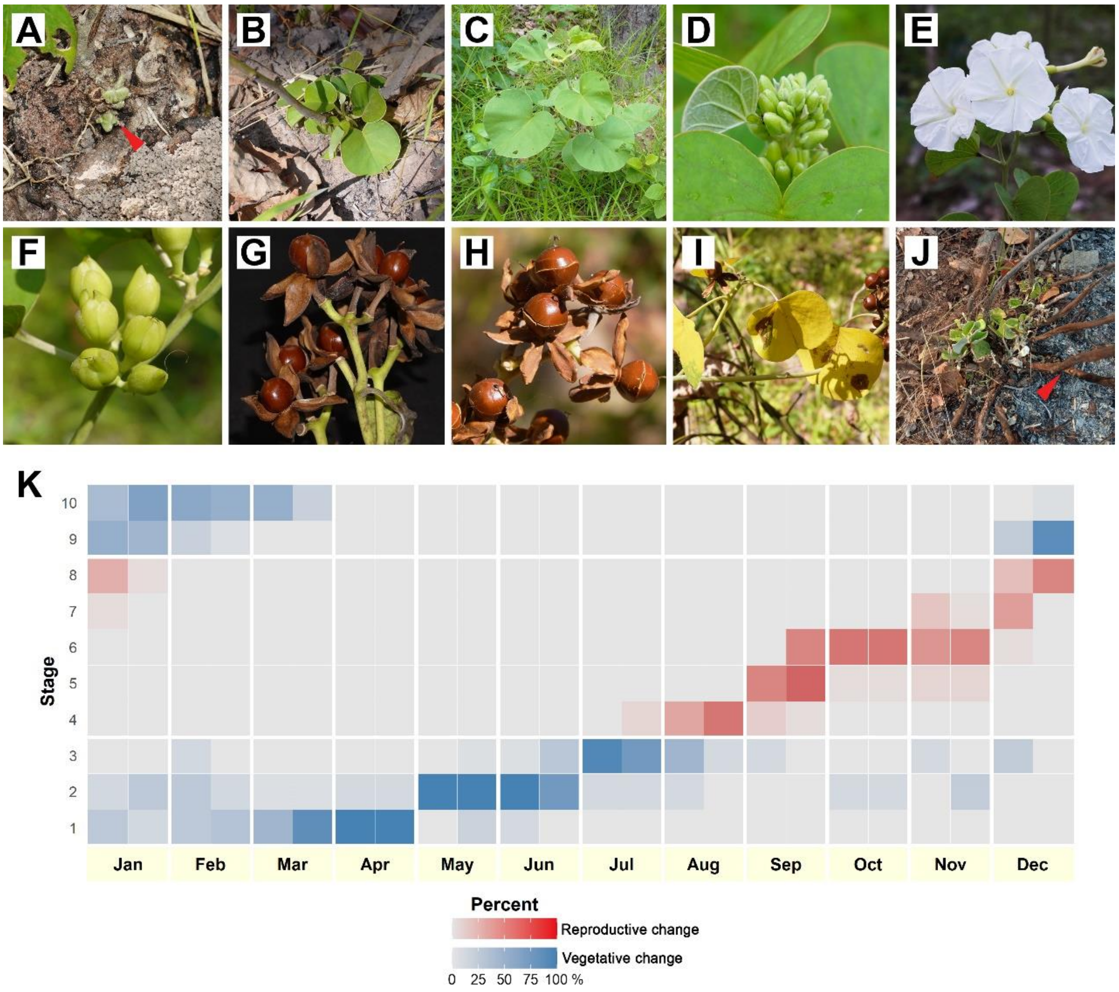


**Additional file 2: Fig. S1.** Growth stages in the annual life cycle of Rivea ornata. (A) Stage 1, leaf bud emergence from rootstock (arrowhead indicates the leaf bud); (B) Stage 2, rosette formation; (C) Stage 3, shoot elongation; (D) Stage 4, flower bud formation; (E) Stage 5, flower blooming; (F) Stage 6, fruit development (green phase); (G) Stage 7, fruit maturation (dry phase); (H) Stage 8, fruit dehiscence; (I) Stage 9, leaf senescence and shedding; (J) Stage 10, stem demise (arrowhead indicates decayed stem); (K) Percentage of plants in each growth stage during each observation round. Blue gradient denotes vegetative stages, and red gradient denotes reproductive stages.

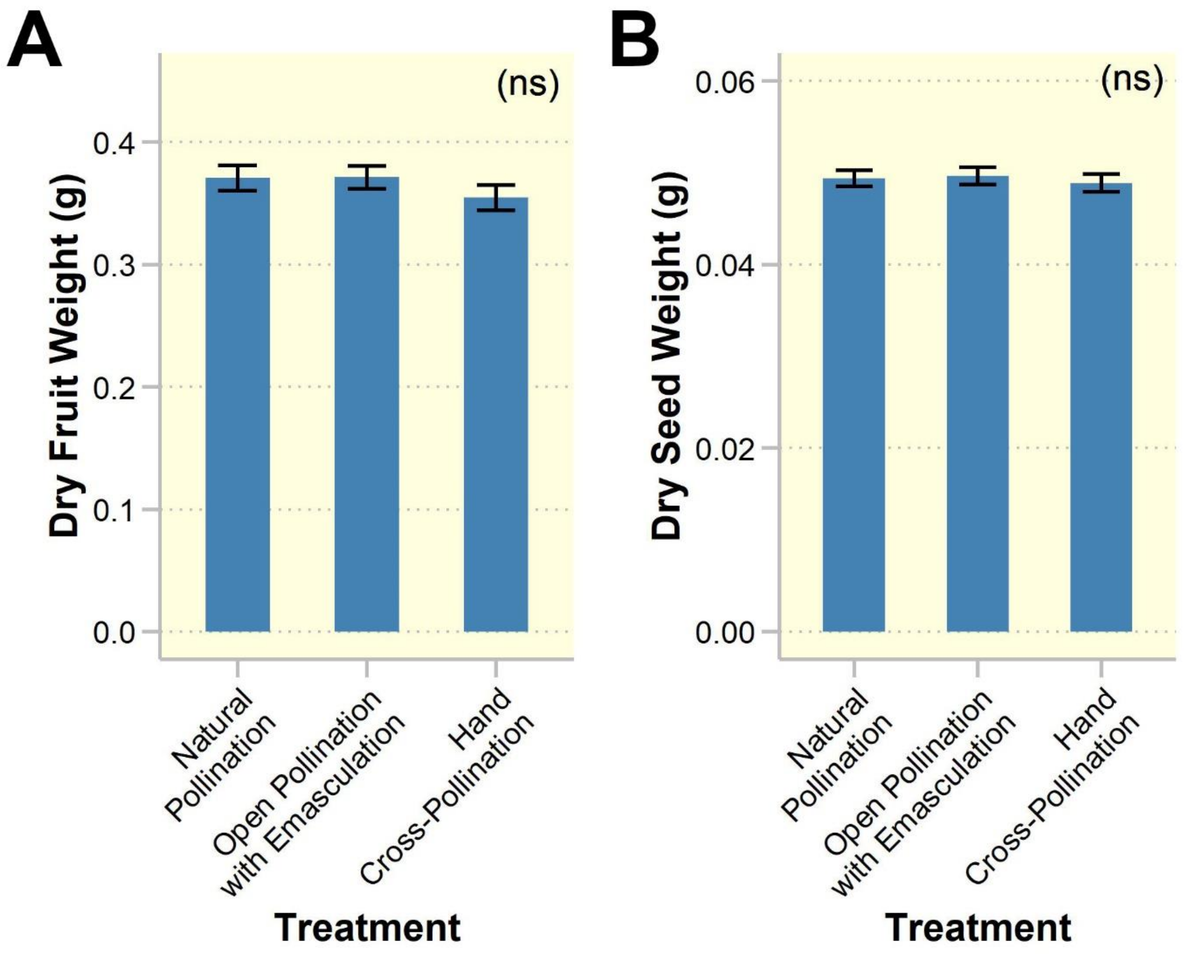


**Additional file 2: Fig. S2.** Additional results from our pollination experiment for the three treatments that successfully set fruit. (A) Dry weight of fruits. (B) Dry weight of seeds. Bars and error bars denote means and standard errors. Abbreviation: ns, not significant.

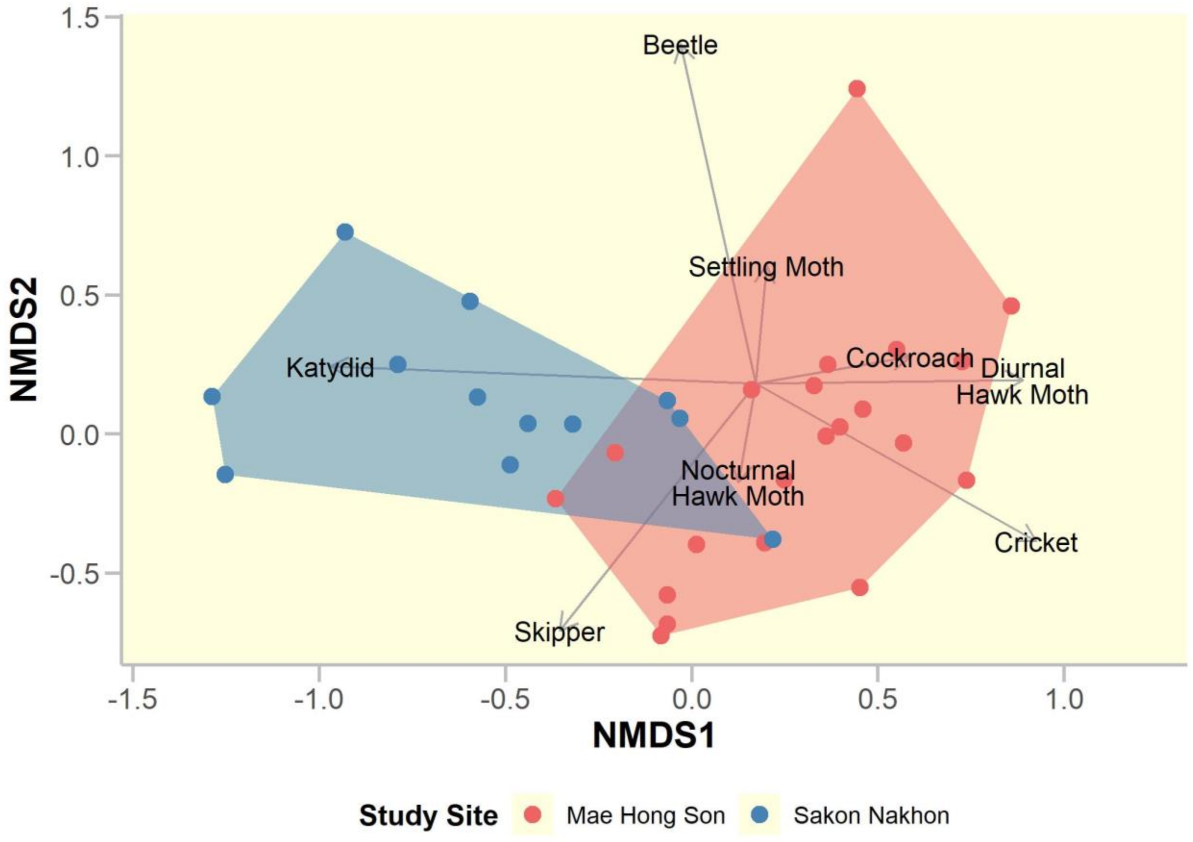


**Additional file 2: Fig. S3.** Visitor composition to *Rivea ornata* flowers at two study sites based on nonmetric multidimensional scaling (NMDS). Dots are observation replicates. Colors denote different study sites; northern population (red), northeastern population (blue).

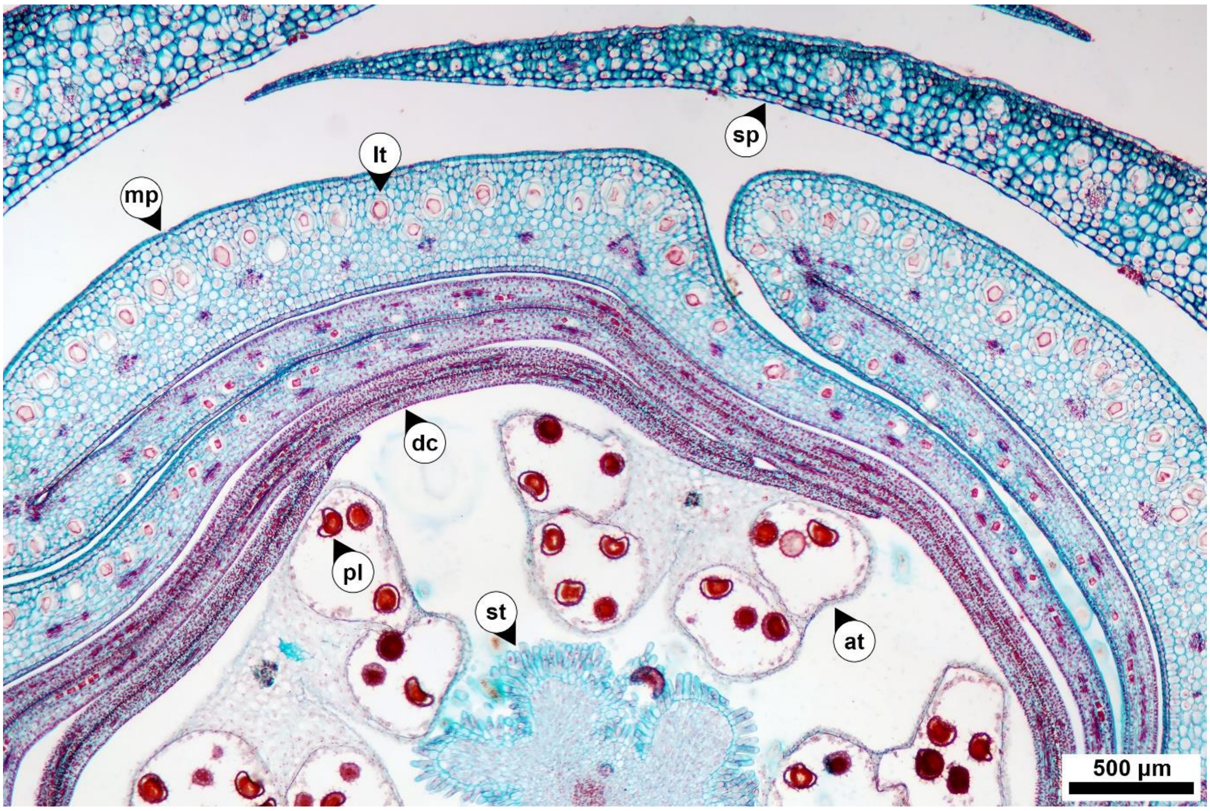


**Additional file 2: Fig. S4.** Floral anatomical characters supportive of the optimal defense hypothesis. The figure shows that size and number of laticifers in the midpetaline bands are greater than those in the delicate areas of the corolla limbs. Anatomical procedure conducted following [19]. Abbreviation: at, anther; dc, delicate corolla limb tissue (i.e., the plica); lt, laticifer; mp, midpetaline band; pl, pollen; sp, sepal; st, stigma. Scale bar 500 µm.
